# Percutaneous management of reperfusion arrhythmias during primary percutaneous coronary intervention: a case report

**DOI:** 10.1186/s43044-021-00158-5

**Published:** 2021-03-25

**Authors:** Hesham Salah El Din Taha, Mirna Mamdouh Shaker

**Affiliations:** Department of Cardiology, Faculty of Medicine, Cairo University, 27 Nafezet Sheem El Shafae St Kasr Al Ainy, Cairo, 11562 Egypt

**Keywords:** Reperfusion arrhythmias, Balloon re-inflation, ST elevation myocardial infarction, Primary percutaneous coronary intervention, Case report

## Abstract

**Background:**

Myocardial reperfusion may cause profound electrophysiological alterations and can lead to serious reperfusion arrhythmias (RA). Management of RA and the accompanying electrical storm that may occur remains a problem. To our knowledge, the role of balloon re-inflation of the infarct-related artery (IRA) has never been addressed as a treatment modality for RA presenting as ventricular tachycardia (VT) with pulse or supraventricular tachycardia (SVT).

**Case presentation:**

Six patients presenting with ST elevation myocardial infarction (STEMI) in the first 12 h, who underwent successful primary percutaneous coronary intervention (PCI), developed RA in the cathlab after restoration of flow in the IRA. The RA was in the form of VT with pulse, except in one patient who had SVT. In four patients, the RA was associated with hemodynamic instability. The mean age of the studied patients was 59.16 ± 7.94 years, and four were males. Coronary artery disease risk factors were prevalent, with four patients being hypertensive, two dyslipidemic, one diabetic, and 2 current smokers. One patient had a history of prior myocardial infarction (MI), and none had a history of congestive heart failure. The coronary angiography showed 100% occlusion of IRA in all patients and 2–3-vessel disease was present in 50%. PCI was successful with restoration of thrombolysis in myocardial infarction (TIMI) 2–3 flow in IRA in all cases. The mean time to revascularization from the onset of chest pain was 4.88 ± 2.68 h. In all cases, balloon re-inflation was successful in terminating the arrhythmias. None of the patients needed direct current cardioversion or anti-arrhythmic drugs for management of the acute arrhythmia.

**Conclusion:**

Balloon re-inflation of IRA was successful in terminating RA that develop in the form of VT with pulse or SVT.

## Background

Timely opening of the infarct-related coronary artery (IRA) by primary percutaneous coronary intervention (PCI) to achieve myocardial salvage continues to be the state-of-the-art treatment for ST elevation myocardial infarction (STEMI) throughout the globe [1]. Despite the clear benefits of reperfusion with improvement of clinical outcomes, reperfusion injury and reperfusion arrhythmias (RA) may be deleterious. Unlike ischemic arrhythmias, RA usually operate through non-reentrant mechanisms [2]. The most common RA are ventricular premature beats and accelerated idioventricular rhythm (AIVR) [3]; however, ventricular tachycardia (VT) and ventricular fibrillation (VF) remain the most important causes of sudden death following spontaneous restoration of antegrade flow [4], hence the importance of prompt management of any serious arrhythmias when they occur.

Currently, the management of serious or sustained RA rests on pharmacologically guided therapy or electrically based therapy (direct current (DC) cardioversion, or pacemaker). Balloon postconditioning was used to ameliorate the reperfusion injury through gradual restoration of flow [5–9]. We here report the novel use of balloon re-inflation of the IRA, in six patients who presented with STEMI within 12 h of chest pain onset, as a method for termination of RA that develop in the form of VT or SVT in the cathlab after successful primary PCI.

## Case presentation

These study cases were reported at the catheterization lab of New Kasr Al Ainy Teaching Hospital, situated in Cairo, Egypt. All six cases presented with STEMI in the first 12 h of chest pain onset. The mean age of the patients was 59.16 ± 7.94 years. Coronary artery disease risk factors were prevalent. The baseline characteristics of the patients are shown in Table 1, including their age, gender, and cardiovascular risk factors.

**Table 1:** Clinical characteristics of patients who developed reperfusion arrhythmias in the form of ventricular tachycardia (with pulse) or supraventricular tachycardia

Case no.	Age (years)	Gender	Prior MI	Prior CHF	Hypertension	Diabetes	Dyslipidemia	Current Smoker
**1**	48	Male	No	No	Yes	No	Yes	Yes
**2**	59	Female	No	No	No	Yes	No	No
**3**	64	Male	Yes	No	Yes	No	No	No
**4**	73	Male	No	No	No	No	No	Yes
**5**	53	Female	No	No	Yes	No	Yes	No
**6**	58	Male	No	No	Yes	No	No	No

*Abbreviations*: *CHF* congestive heart failure, *MI* myocardial infarction

All patients received a loading dose of two antiplatelet therapies before PCI and unfractionated heparin was given during the procedure. After consent for invasive management had been obtained, the interventional procedures were performed using the femoral approach. The primary PCI was done using 6-F guiding catheters.

### Case 1

A 48-year-old male patient presented to the emergency department (ED) with typical chest pain and an ECG revealing anterior STEMI. He was a smoker with a history of hypertension and dyslipidemia. The coronary angiography revealed 100% occlusion of the LAD. Primary PCI was done to the IRA with TIMI 3 flow and a time to revascularization 2.5 h. The patient developed VT with hemodynamic instability, and while preparing for DC cardioversion, the operator decided to re-inflate the balloon at the site of the lesion for 1 min as a possible way to terminate the RA, and the VT was actually terminated and did not recur.

### Case 2

A 59-year-old diabetic female patient presented to the ED with typical chest pain and an ECG showing postero-lateral STEMI. The coronary angiography revealed two-vessel disease with total occlusion of the LCX. Using a 6-F guiding catheter, primary PCI was done to the LCX with TIMI 3 flow and a time to revascularization 3.3 h. The patient developed VT but was hemodynamically stable, and while preparing for DC cardioversion, the operator re-inflated the balloon at the site of the lesion for 1 min. The VT was terminated and did not recur.

### Case 3

A 64-year-old hypertensive male patient, with a history of prior MI, underwent primary PCI to the LAD after being diagnosed with anterior STEMI with an excellent outcome and TIMI 3 flow. The time to revascularization in this case was 9.4 h. Following successful revascularization, the patient developed VT with hemodynamic instability, which was also terminated by a 1-min balloon re-inflation of the IRA. The arrhythmia recurred again on balloon deflation. Re-inflating the balloon again for 1 min terminated the arrhythmia which did not recur.

### Case 4

In this case, a 73-year-old male patient, a smoker, presented with an anterior STEMI. The coronary angiography revealed a three-vessel disease with the totally occluded LAD being the IRA. PCI to the LAD was done with a TIMI 2 flow and time to revascularization 2.1 h. The patient then developed VT with no vital instability. The RA was successfully terminated by a 1-min balloon inflation at the culprit lesion of the LAD.

### Case 5

This is the case of a 53-year-old female patient that was known to be hypertensive and dyslipidemic. She presented to the ED department with typical chest pain and was diagnosed to have an inferior STEMI associated with RV infarction. The angiography revealed a two-vessel disease with the RCA being the culprit artery. After successful primary PCI to the RCA, the patient developed SVT and was hemodynamically unstable. Similar to the previous cases, termination of the RA was attempted by balloon re-inflation of the RCA and was successful. In this case, the time to revascularization was 7.5 h.

### Case 6

In this case, a 58-year-old hypertensive male patient underwent primary PCI to the RCA after being diagnosed with an inferior STEMI with TIMI 3 flow and time to revascularization 4.5 h. Following successful revascularization, he developed VT and became vitally unstable. As before, the arrhythmia was successfully terminated by balloon re-inflation of the IRA.

The procedural details of the six cases including the mode of presentation, coronary angiographic characteristics, type of reperfusion arrhythmia, and effects of balloon re-inflation on reperfusion arrhythmias are all described in Table 2.

**Table 2:** Mode of presentation, coronary angiographic characteristics, type of reperfusion arrhythmia, and effects of balloon re-inflation on reperfusion arrhythmias

Case no.	Presentation	Time to revascularization (hours)	Hemodynamic instability	Number of diseased coronary vessels	IRA	% Stenosis of IRA	TIMI flow (post-angioplasty)	RA	RA termination on balloon re-inflation
**1**	Anterior STEMI	2.5	Yes	1-VD	LAD	100%	3	VT	Successful
**2**	Postero-lateral STEMI	3.3	No	2-VD	LCX	100%	3	VT	Successful
**3**	Anterior STEMI	9.4	Yes	1-VD	LAD	100%	3	VT	Successful
**4**	Anterior STEMI	2.1	No	3-VD	LAD	100%	2	VT	Successful
**5**	Inferior + RV STEMI	7.5	Yes	2-VD	RCA	100%	3	SVT	Successful
**6**	Inferior STEMI	4.5	Yes	1-VD	RCA	100%	3	VT	Successful

*Abbreviations*: *IRA* infarct-related artery, *LAD* left anterior descending artery, *LCX* left circumflex artery, *RCA* right coronary artery, *RA* reperfusion arrhythmias, *RV* right ventricular, *STEMI* ST elevation myocardial infarction, *SVT* supraventricular tachycardia, *VT* ventricular tachycardia

The coronary angiography showed 100% occlusion of IRA in all patients and 2–3-vessel disease was present in 50%. PCI was successful with restoration of thrombolysis in myocardial infarction (TIMI) 2–3 flow in IRA in all cases. The mean time to revascularization from onset of chest pain was 4.88 ± 2.68 h. Following successful revascularization, five patients developed VT with pulse and one patient had SVT. The RA was associated with hemodynamic instability in four patients. In all cases, balloon re-inflation was successful in terminating the arrhythmias. None of the patients needed DC cardioversion or anti-arrhythmic drugs for management of the acute arrhythmia.

## Discussion

In patients presenting with STEMI, infarct size can be limited by early myocardial reperfusion via primary PCI, thereby preserving left ventricular systolic function and improving clinical outcome. However, the full benefits of myocardial reperfusion may be limited by the dramatic ionic and metabolic disturbances that can induce myocardial stunning, no-reflow phenomenon, RA, or lethal reperfusion injury [10–14]. Consequently, reperfusion may paradoxically enhance myocardial injury, finally contributing to 50% of the final MI size [14].

Reperfusion injury results from several complex and interdependent mechanisms that involve altered myocardial metabolism with the production of ROS, mishandling of intracellular calcium, microvascular and endothelial cell dysfunction, platelet, neutrophil, and complement activation [15].

Arrhythmias occurring during the ischemia/reperfusion period may be due to ischemia, no-reflow after opening of an IRA, or due to reperfusion injury leading to RA. The underlying mechanism of each differs although the type of arrhythmia may be indistinguishable. Unlike ischemic arrhythmias which are usually reentrant in nature, RA operate mainly through non-reentrant mechanisms such as abnormal or enhanced automaticity and triggered activity due to afterdepolarizations occurring as a result of intracellular calcium overload leading to spontaneous calcium oscillations [2, 16].

Previous studies using retrospective registry data showed a high incidence of arrhythmias following intervention, with new-onset AF ranging from 6 to 28%, non-sustained VT in 7–13%, high-degree AV block in 5–10%, sinus bradycardia in 7–16%, sinus arrest (≥ 5 s) in 5%, sustained VT in 3–6%, and VF in 3–6% [17].

In another study on 503 patients with STEMI, the arrhythmias and conduction disturbances occurring from arrival at the catheterization laboratory to 90 min after primary PCI were registered. The most common arrhythmias observed during primary PCI were AIVR in 42%, sinus bradycardia in 28%, and non-sustained VT in 26% [18].

Studies on RA in patients during the first 24 h after reperfusion using thrombolytic treatment revealed a prevalence of ventricular premature complexes and couplets of nearly 100%. Ninety percent of patients had an average of eight runs of AIVR per hour per patient, and VT in 23% of the patients had an average of two runs per hour per patient during the first 24 h after reperfusion [19].

Mehta et al. [20] published data on the incidence of major arrhythmia (VT and VF) in 5745 patients treated with primary PCI in the APEX AMI trial. They found that VT/VF occurred in 5.7% of patients, usually before catheterization was completed (64%).

In other studies, ventricular arrhythmias requiring electrical counter-shock, including VF and rapid sustained VT, occurred in 1.5 to 2.9% of patients undergoing percutaneous transluminal coronary angioplasty (PTCA) [21–23].

Different factors may explain the differences in the incidence of RA including differences in the time frame used for defining RA which ranged from 90 min [18] up to 48 h [20], as well as how reperfusion was achieved and how rapid was this done.

The earlier the revascularization therapy is initiated and the more rapidly the reperfusion is achieved, the more is the frequency of RA. Therefore, RA was more common in pre-hospital thrombolysis compared to in-hospital lytic therapy, as shown in a study on 5469 patients that was conducted by the European Myocardial Infarction Project Group [24]. Furthermore, primary PCI is more often associated with RA compared to lytic therapy in acute MI, as proved in a randomized clinical study [25].

The widespread availability of revascularization therapy and the increased use of beta-blockers have decreased the incidence of sustained VT and VF occurring within 48 h of the onset of an acute coronary syndrome (ACS) over the past decade [26].

An advantage of the current study is that the arrhythmias occurred immediately after opening of IRA and resumption of flow, with the patient monitored in the cathlab, in a setting and time frame consistent with the occurrence of RA and not induced by ischemia.

Some of the RA are self-limited and do not need any intervention; however, others are sustained and are managed either pharmacologically or electrically (DC cardioversion or pacemaker) based on the type of arrhythmia.

In our study, six patients developed RA in the form of VT or SVT during the primary PCI procedure after restoring flow in the IRA. In case 1, the patient developed VT with hemodynamic instability, and while preparing for DC cardioversion, the operator decided to re-inflate the balloon at the site of the lesion for 1 min as a possible way to terminate the RA, and the VT was actually terminated and did not recur. In case 3, while VT was also successfully terminated by balloon re-inflation, the arrhythmia recurred again on balloon deflation. Re-inflating the balloon again for 1 min terminated the arrhythmia which did not recur. The success rate of this cost-free technique was 100% in all six patients (Table 2), and the effect was immediate within the 1-min balloon inflation. None of the patients needed DC cardioversion or anti-arrhythmic drugs for management of the acute arrhythmia obviating the patient from the possible pain and skin burns associated with the cardioversion and the possible side effects of medications.

In concordance with our results, although on a different type of arrhythmia, Grech and Ramsdale [27], in their single case report on a patient who developed AIVR following successful right coronary artery recanalization by primary PTCA, could achieve sinus rhythm on re-inflating the PTCA balloon. With each balloon deflation, idioventricular rhythm developed again with associated hypotension. A 100-mg intravenous bolus of lignocaine did not restore sinus rhythm, which spontaneously returned 20 min after the last balloon deflation.

Our results may be a clinical parallel to an experimental study on isolated Langendorff-perfused rat hearts which demonstrated that postconditioning by a single brief episode of global ischemia can effectively terminate persistent reperfusion-induced VF and convert it into a normal rhythm. Regular beating was maintained by all postconditioned hearts during the subsequent reperfusion. However, these experimental data were only done on reperfusion-induced VF [9].

Previous attempts to minimize reperfusion injury using mechanical ischemic postconditioning in which brief, intermittent episodes of inflation and deflation were done by angioplasty balloon starting within 1 min after reperfusion [6–9] demonstrated in several small-sized studies to be efficacious during the primary PCI procedure to further improve the prognosis of STEMI beyond myocardial reperfusion [28–31].

To our knowledge, this is the first report which shows the capability of balloon re-inflation in terminating RA in the form of VT (with pulse) and SVT during primary PCI after successful reperfusion. The mechanism of successful termination may be through temporary interruption of reperfusion, giving a chance for the accompanying electrical and biochemical chaos to ease, leading to reduction of oxygen free radicals, modulation of calcium overload, and correction of acidosis [6].

### Limitations

The present case study is not without limitations. First, a significant limitation is the relatively small number of patients included, and being a single-center study. However, these case reports, which are to our knowledge reported for the first time, should be considered hypothesis-generating and encourage larger-scale studies. Second, some of the RA are self-limited, and it is possible that even without balloon inflation some of these arrhythmias would have been spontaneously terminated. However, the time relation of balloon deflation and occurrence of arrhythmias and re-inflation and disappearance of the arrhythmia in a controlled cathlab setting favors the effect of balloon inflation on the termination of arrhythmias. A third limitation is that the technique of balloon re-inflation to terminate RA was used in only specific types of RA and was not attempted in the others.

## Conclusions

Reperfusion arrhythmias are common during primary percutaneous coronary interventions after successful reperfusion. Immediate management using balloon re-inflation of the infarct-related artery during the procedure was successful in terminating ventricular tachycardias (with pulse) and supraventricular tachycardias. Larger-scale studies are needed to confirm these findings, and a trial to use on other life-threatening reperfusion arrhythmias is warranted.

## Data Availability

The dataset supporting the results and conclusions of this article will be available from the corresponding author on request.

## Abbreviations

ACS: Acute coronary syndrome
AF: Atrial fibrillation
AIVR: Accelerated idioventricular rhythm
AV: Atrioventricular
DC cardioversion: Direct current cardioversion
ED: Emergency department
IRA: Infarct-related artery
LAD: Left anterior descending artery
LCX: Left circumflex artery
MI: Myocardial infarction
PCI: Percutaneous coronary intervention
PTCA: Percutaneous transluminal coronary angioplasty
ROS: Reactive oxygen species
RA: Reperfusion arrhythmias
RCA: Right coronary artery
RV: Right ventricular
STEMI: ST elevation myocardial infarction
SVT: Supraventricular tachycardia
TIMI: Thrombolysis in myocardial infarction
VF: Ventricular fibrillation
VT: Ventricular tachycardia
